# An insect-inspired bionic sensor for tactile localization and material classification with state-dependent modulation

**DOI:** 10.3389/fnbot.2012.00008

**Published:** 2012-08-02

**Authors:** Luca Patanè, Sven Hellbach, André F. Krause, Paolo Arena, Volker Dürr

**Affiliations:** ^1^Dipartimento di Ingegneria Elettrica Elettronica e Informatica, University of CataniaCatania, Italy; ^2^Lehrstuhl für Biologische Kybernetik, Fakultät für Biologie, Universität BielefeldBielefeld, Germany; ^3^Cognitive Interaction Technology – Center of Excellence, Universität BielefeldBielefeld, Germany

**Keywords:** bionic sensor, forward model, insect antenna, material classification, spiking network, tactile localization, tactile sense

## Abstract

Insects carry a pair of antennae on their head: multimodal sensory organs that serve a wide range of sensory-guided behaviors. During locomotion, antennae are involved in near-range orientation, for example in detecting, localizing, probing, and negotiating obstacles. Here we present a bionic, active tactile sensing system inspired by insect antennae. It comprises an actuated elastic rod equipped with a terminal acceleration sensor. The measurement principle is based on the analysis of damped harmonic oscillations registered upon contact with an object. The dominant frequency of the oscillation is extracted to determine the distance of the contact point along the probe and basal angular encoders allow tactile localization in a polar coordinate system. Finally, the damping behavior of the registered signal is exploited to determine the most likely material. The tactile sensor is tested in four approaches with increasing neural plausibility: first, we show that peak extraction from the Fourier spectrum is sufficient for tactile localization with position errors below 1%. Also, the damping property of the extracted frequency is used for material classification. Second, we show that the Fourier spectrum can be analysed by an Artificial Neural Network (ANN) which can be trained to decode contact distance and to classify contact materials. Thirdly, we show how efficiency can be improved by band-pass filtering the Fourier spectrum by application of non-negative matrix factorization. This reduces the input dimension by 95% while reducing classification performance by 8% only. Finally, we replace the FFT by an array of spiking neurons with gradually differing resonance properties, such that their spike rate is a function of the input frequency. We show that this network can be applied to detect tactile contact events of a wheeled robot, and how detrimental effects of robot velocity on antennal dynamics can be suppressed by state-dependent modulation of the input signals.

## Introduction

The sense of touch is a prime source of information about object features within the near-range environment. Many animals carry actively moveable tactile sensors with which they explore and sample the ambient space (Prescott et al., 2011). Of these, the whiskers of mammals (Diamond et al., 2008; Mitchinson et al., 2011) and the antennae (or feelers) of insects and crustaceans (Staudacher et al., 2005) are amongst the most elaborate sensory structures for active tactile exploration. Thus, it is not surprising that a number of artificial tactile sensing systems have been developed that capture important aspects of mammal whiskers or insect antennae.

Pioneering studies on contact sensing with actuated passive probes were loosely inspired by whiskers or antennae. They either used torque or vibration sensors at the base of an otherwise non-sensorized beam to infer contact location from bending (e.g., Tsujimura and Yabuta, 1992; Kaneko et al., 1998) or resonant behavior of the beam (e.g., Ueno et al., 1998). More recently, whisker-inspired sensor arrays have been developed for shape recognition in a stationary system (Solomon and Hartmann, 2006), but also for active exploration of objects by mobile robot platforms (e.g., Pearson et al., 2007, 2011). Insect-inspired applications with active feelers include tactually mediated decision-making for climbing *versus* tunnelling in a cockroach-inspired robot (Lewinger et al., 2005). All of these approaches have in common that the probe itself is a non-sensorized beam, and that tactile information is gathered by active exploration of the environment.

Mammal whiskers are hairs and, as such, are well-modeled by a non-sensorized beam held by a sensorized shaft. In contrast, insect antennae are multimodal and highly sensorized limbs of the head. As limbs, they contain at least two joints actuated by muscles, and may carry thousands of individual sensors in modalities as different as smell, taste, hygroreception (for humidity), thermoreception (for temperature), and touch (reviewed by Staudacher et al., 2005). Bionic analogs of insect antennae therefore should be sensorized probes. To date, the most elaborate insect-inspired sensorized antennae are passive, at least in the sense that they do not actively sample the space around the robot “body.” As yet, they have been applied successfully in a tactile course-control paradigm inspired by wall-following behavior of cockroaches (Lee et al., 2008). The underlying principle is to infer the distance to the wall from a series of bending-sensitive elements (Cowan et al., 2003; Lamperski et al., 2005) that may even be tuneable in order to account for different functional properties along the probe (Demir et al., 2010).

Here, we propose an insect-inspired active tactile sensor that complements the approaches mentioned above by considering a sensorized and actively moveable probe suitable for tactile exploration on a mobile robot platform. The present paper has three objectives: (i) first it will review the measurement principle underlying vibration-based tactile localization (Lange et al., 2005, patented by Lange and Reimann, 2005) and material classification (Dürr et al., 2007). (ii) Second, it will demonstrate the implementation of this measurement principle by means of Artificial Neural Networks, ANNs (Hellbach et al., 2010), including considerations of the resource-performance trade-off (Hellbach et al., 2011). (iii) Thirdly, it will demonstrate the applicability of the system on a mobile robot, using a spiking neural network (Arena and Patanè, 2012) allowing for state-dependent modulation for separating self-induced stimulation from external stimulation.

The latter concerns a general problem of sensory systems in moving bodies, and also concerns technical applications in which self-motion of a system interferes with and potentially confounds the analysis of sensor readings. Whereas in animals and humans, the mechanisms underlying the separation of self-induced and external stimulation are often summarized by the terms *corollary discharge* and/or *efference copy* (different variants of such mechanisms are reviewed by Crapse and Sommer, 2008), in technical systems, they typically involve the definition of a forward model (see Karniel, 2002, for distinction of three variants of forward models). In more general terms, such mechanisms not only concern dealing with self-induced sensory input, but also predicting the behavior of a dynamical system in general, including its motor output. Several studies have addressed the analogies of predictive forward models in physiological and technical systems, (e.g., Miall and Wolpert, 1996; Mehta and Schaal, 2002; Schröder-Schetelig et al., 2010), including the putative role of forward models in insect neurobiology (Webb, 2004). In the context of active tactile sensing in insects, Gebhardt and Honegger (2001) described descending interneurons that are sensitive to antennal movement and whose responsiveness changes in the presence of antennal motor activity. Although the underlying mechanism has not been identified in the antennal system, it is reminiscent of a well-known mechanism in the auditory pathway of the same insect species (Poulet and Hedwig, 2002, reviewed by Poulet and Hedwig, 2007).

In analogy to the mechanism discovered by Poulet and Hedwig, the present study implements a simple forward model in the form of state-dependent modulation which, according to the classification scheme of Crapse and Sommer (2008), belongs to the lower-order corollary discharge mechanisms for “central control of sensation.” The core of the model is a spiking neural network consisting of a sensory array of resonate neurons. This sensory array extracts the relevant information related to contact events registered by the antenna. During self-motion of the robot, the motor speed command, i.e., the motor activity, is used to predict the strength of modulation of the input to the sensory array, thus adapting the sensory processing to the current “behavioral state.” We show how such activity- or state-dependent modulation allows separation of self-induced antennal stimulation from stimulation related to active touch.

## Materials and methods

### Bionic antenna

**Figure 1:**
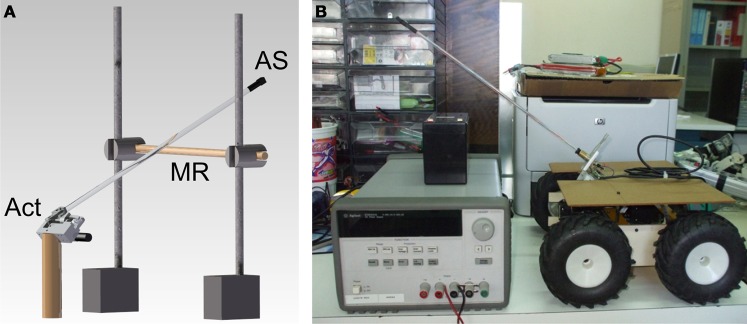
**Bionic antenna and robotic platform. (A)** Stationary setup for experiments on tactile localization and material classification. The probe consisted of a polyacrylic beam with a distal acceleration sensor, AS. It was actuated by a custom-built two-axis actuator platform, Act, using DC-motors and linkages. During experiments, the probe was moved up-and-down and hit a cylindrical metal rod, MR that was made of different materials and located at varying distances. **(B)** For test experiments on state-dependent modulation, the probe was mounted onto a pan-tilt unit on a wheeled robot.

The bionic feeler used throughout this study (Figure 1) captured three major characteristics of the stick insect antenna: (i) it is a beam-like structure actuated by two rotary joints (Dürr et al., 2001); (ii) it is compliant but also stiff enough to maintain its shape during self-motion (Dirks and Dürr, 2011); and (iii) it is vibration-sensitive (Westmark and Dürr, 2009). The probe consisted of a 33 cm polyacrylic tube that carried a distal two-axis acceleration sensor (Analog Devices ADXL210E). It was mounted to the actuator platform *via* a threaded electric plug connector, allowing simple exchange of probes without affecting the actuator platform. For the experiments on tactile localization and material classification, the actuator platform consisted of two orthogonal axes, each one driven by a 6V DC motor (Faulhaber 1331T 006SR). The linkage was designed to mimic the action range of the stick insect antenna, amounting to 90° in the vertical range, centered 10° above the horizon, and to 80° horizontal range centered 40° to the side. Two rotary position sensors (muRata SV01A potentiometers) monitored the orientation of the probe, thus supplying the two angles required for representing 3D contact location in a spherical coordinate system. The third dimension required for this representation, i.e., distance along the probe, was to be inferred from the sensor readings of the acceleration sensor (see below). For initial experiments, antennal movement was controlled by manual switching of a voltage source, and sensor readings were registered using an AD converter system (CED 1401 power, controlled by Spike2, Cambridge Electronics Design). For acquisition of larger data-sets, as necessary for ANN training, antennal movement control as well as sensor read-out were implemented on an embedded system (ATMEL AT90CAN128), with the raw sensor signal being available *via* RS232C for further processing in Matlab (The Mathworks). Angular positioning of the probe was limited by slack in the motors and amounted to approximately 7° (5 mm at the distal end of the probe). Total length of the feeler was 413 mm. Total weight was 175 g, including both motors.

### Pre-processing, detection of contact events, and parameter extraction

**Figure 2:**
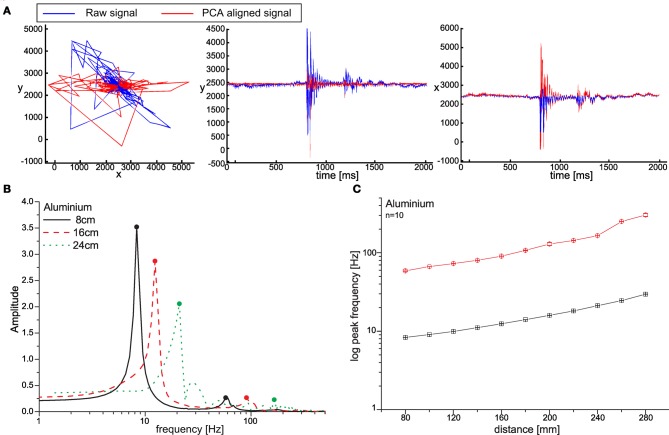
**FFT-based parameter extraction. (A)** The raw sensor signal (blue) consisted of two channels, which measured the acceleration of the antenna in two dimensions (*x* and *y*, plotted in arbitrary units). Since the antenna could hit an obstacle in an arbitrary angle, the signal needed to be aligned for further processing. This was done using principal component analysis (PCA) (red curve). PCA determined a new coordinate-system, in which the first axis contained the maximum variance of the data. The dimension with the higher variance was selected and transferred into Fourier frequency space. Contact distance was encoded by oscillation frequencies. **(B)** FFT amplitude spectra of three single contacts with an aluminium rod, taken at 8, 16, and 24 cm distance. As contact distance increased, the low-frequency peak decreased in amplitude and shifted to higher frequencies. Multiple high-frequency modes could occur, but only the largest mode beyond 55 Hz was analysed further. **(C)** Peak frequencies of both frequency ranges increased exponentially with distance (*n* = 10, means ± SD, note that error bars are within symbols); red: high frequency component; black: low frequency component.

The distal acceleration sensor provided readings corresponding to two orthogonal dimensions, such that the actual oscillation of the antennal tip was projected onto the corresponding dimension vectors of the sensor. Because of this, sensor readings depended on the orientation of the sensor with respect to the antennal movement direction at the time of a contact event. To align the rotated oscillation with a single axis, principle component analysis, PCA, was applied. PCA computes a set of eigenvectors which are oriented with respect to the principal axes of the data distribution. The matrix of eigenvectors *E* can be used directly as an affine transform matrix applied to the data: *X*_rotated_ = *E* · *X*. Here, the first dimension of the rotated data *X*_rotated_ contained the part of the data with the largest variance. For all experiments reported in this paper, only this part was used for further processing (Figure 2A).

As a first test for proof of principle in contact localization and material classification, we constrained the movement to the vertical axis and analysed sensor readings upon contact with one of two horizontal test rods (Figure 1A) made of either aluminium (Ø = 11.8 mm) or wood (Ø = 9.6 mm). For subsequent detailed analysis of performance in classification of multiple materials, test rods were made of the following eight materials: aluminium, stainless steel, wood, copper, brass, polyoxymethylene (POM), polyvinylchloride (PVC), and acrylic glass. In these experiments, all test rods were 12 mm in diameter. Whenever an impact of the antenna on an obstacle occurred (contact event), the acceleration sensor recorded the damped harmonic oscillation of the antennal tip. Thus, for processing information relevant to contact events it was necessary (i) to detect the corresponding damped oscillation and (ii) to retrieve the relevant information for describing the properties of the oscillation recorded. In the stationary system, the contact could be detected easily and reliably by means of a simple threshold for the acceleration. For detecting the end of the oscillation, the local maxima over time were considered. The end point was defined as the time at which the amplitude of these maxima decreased below 10% of the maximum amplitude. Only the data within the window between the detected start and end points was processed further. In a first step, the mean signal amplitude was subtracted. Next, the frequency content of the damped oscillation was determined by means of a Fast Fourier Transform (FFT, using FFT algorithms of Matlab or MathCad, Adept Scientific). The result of the FFT was used in two different variants for further processing (see neural network section “Neural Network for Localization and Material Classification”). In case of the “parametric variant” the FFT result was used to extract six parameters that captured the most important signal properties. In the “FFT variant,” the entire amplitude spectrum of the FFT was used without further pre-processing. Whereas the parametric variant was used for proof of principle and for sensitivity analysis of distinct parameters, the FFT variant was used to find the best performance possible in case of maximum information available.

As the typical frequency spectrum of a contact-related signal contained two distinct peaks (Figure 2B), the purpose of parameter extraction was to describe the two corresponding frequency components in terms of their amplitude, A; frequency, F; and decay time constant, τ. For contact events along the proximal three quarters of the probe, it was sufficient (i) to divide the FFT spectrum in two parts (using 55 Hz as a fixed boundary between low- and high-frequency components), (ii) to determine the peak frequency, F, within each part of the spectrum, and (iii) then reconstruct the signal corresponding to these frequency components, only (using inverse FFT-algorithms of Matlab or MathCad). The peak frequencies depended on the contact location following a logarithmic function (Figure 2C).

Damping was quantified by estimating the decay time constant from the local extreme points of the damped oscillation. The most satisfying results were obtained with the following algorithm: after decomposition of the recorded signal into a pair of low- and high-frequency components as described above for steps (i) to (iii), (iv) local extreme points were extracted with a minimum interval (e.g., 3 ms) and minimum absolute amplitude (e.g., 15 mV). Next, (v) the amplitudes of the extreme points were rectified and log-transformed. Finally, (vi) a linear regression yielded the amplitude, A, and time constant, τ, of a first-order exponential decay function.

### Neural network for localization and material classification

ANN were programmed and trained either by use of custom-written software (for proof of principle) or by use of the Neural Networks Toolbox of Matlab. For distance estimation and material classification tasks, we used simple feed-forward ANNs, either single- or multi-layered perceptrons. For proof of principle using the parametric variant, a single ANN was used for combined distance estimation and the distinction of two materials (wood *versus* aluminium). Several different combinations of input parameters were tested, as will be elaborated in the results section. In the FFT variant, separate networks were used for localization and material classification, although in principle, both could be combined into a single ANN. Input to the FFT variants was either the entire Fourier spectrum (509 frequency components) or the result of the dimension reduction algorithm (see below). Best results for distance estimation were obtained using a 3-layered ANN with 20 neurons for the first hidden layer and 5 for the second layer. For material classification a two-layered network with 51 neurons in the hidden layer was sufficient. All networks were trained by use of a gradient descent method.

For reducing the input dimension in a data-driven manner, we used non-negative matrix factorization (NMF, Lee and Seung, 1999). Two other algorithms that, like NMF, also compute a vector basis transformation were tested too (PCA and Partial Least Squares, Schwartz et al., 2009), but will not be presented here. The main reason for using NMF was that it produces a set of basis vectors that, when applied to the input, has similar computational properties as a set of band-pass filters. Most importantly, it attenuates or amplifies each input component with positive scaling factors only. This property makes the solution physiologically plausible, as it captures the frequency-selective attenuation which is typical for sensory processing (e.g., Braddick et al., 1978). It arises due to the constraint of the NMF algorithm, allowing non-negative basis vector components only. In contrast, algorithms such as PCA and PLS produce basis vectors with negative components and, therefore, do not capture the computational properties of band-pass filters.

### Robotic platform

The robotic system used for the implementation of the feed-forward strategies, consisted of a custom-built, dual-drive roving platform equipped with a pan-tilt actuated bionic-antenna, as shown in Figure 1A. The pant-tilt unit consisted of two Dynamixel motors (i.e., High-performance networked actuators for robots RX-64, http://www.robotis.com/xe/) controlled through an RS485 serial bus. The same motors were used also to actuate the four robot wheels. The low-level distributed control system was based on three main boards: (i) an 8-bit Atmega-based board used to handle the ADXL321 sensor; the *x* and *y* signals coming from the sensor were sampled with a 10-bit ADC at a sampling rate of 1 kHz, before being transferred to a PC *via* a USB connection; (ii) a 128-bit Atmega-based board was used to control the pan-tilt system of the antenna; in the simplest case, the two motors followed a limit cycle with a period of two seconds; (iii) the main board, based on a 128-bit Atmega microcontroller, was used to control the movements of the roving platform and received commands from or exchanged data with a remote PC through a wireless connection.

### Spiking neural model

**Figure 3:**
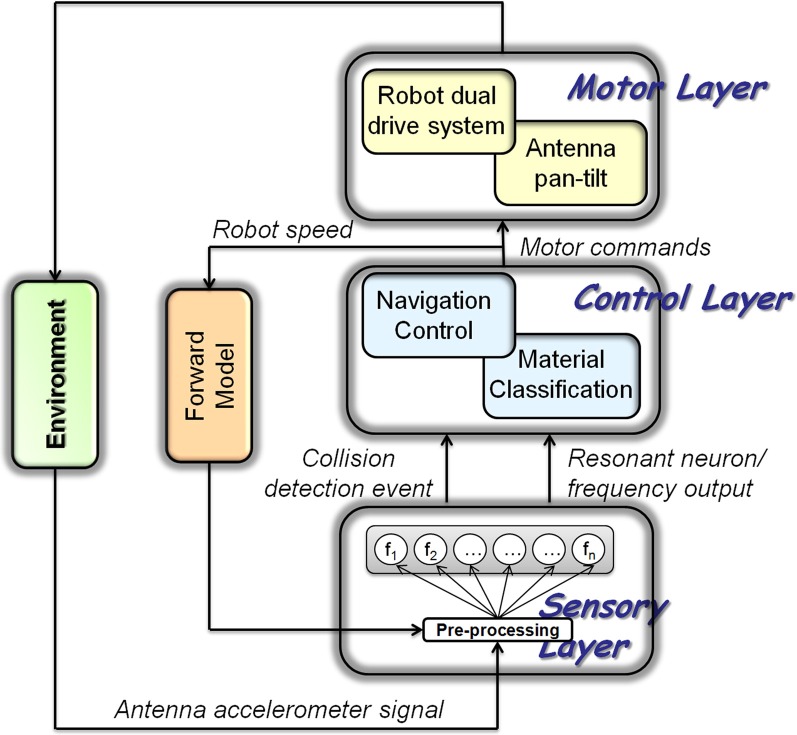
**Bock diagram of the control architecture, designed for the integration of the bionic antennae in a roving platform.** The neural network was characterized by multiple layers: (i) A Sensory Layer used for sensory pre-processing and extraction of the frequency spectrum of the acquired signal, based on an array of resonate-and-fire neurons; (ii) a Control Layer where a high-level navigation controller could be introduced together with the material classification network, and (iii) a Motor Layer, used to actuate both robot wheels and the pan-tilt unit of the antenna. Details of the Motor Layer and the Navigation Control unit of the Control Layer are beyond the scope of this paper. The presence of a forward model for self-motion compensation has been considered by using a sensory gating strategy. The commands for speed were used to modify the pre-processing of the antennal acceleration sensor in oder to compensate for self-induced sensor readings.

The sensory data acquired with the robotic platform was processed by a spiking neural network model. The model combined linear sensory arrays of spiking neurons and a central pattern generator (CPG) model for driving the pan-tilt unit (Figure 3). After a pre-processing stage, the sensory information was fed into a one-dimensional array of spiking sensory neurons. Through plastic synapses, their output was then conveyed to the motor neurons that control the muscle/motor system. The scheme proposed in Figure 3 refers to the general case in which the robot is equipped with two antennae, even though in the experiments described here, sensor data were acquired from a single antenna only.

As described above, the sensory information acquired with the two-axis accelerometer was pre-processed using PCA and normalized. The pre-processed signal was used as input current for a series of resonate-and-fire neurons. Each neuron was tuned to resonate at a specific frequency in the interval from 7 to 30 Hz. The high-frequency band necessary for material classification was not used in the robot experiments, as we concentrated on distance estimation only The array of resonate neurons used to process the sensory signals for each antenna represented a linear sensory array encoding the contact location along the antenna. Whereas neuron *f*_1_ provided information about the lowest frequency component (dominated by self-stimulation), neurons *f*_2_–*f*_n_ provided information about increasingly higher frequency components and were associated with contact detection of increasingly distal locations along the probe (with increasing *n* in *f*_*n*_). The array of resonate-and-fire neurons provided input to the control layer, of which only one part will be explained in this paper (Material Classification). Whereas the Navigation Control unit determined the appropriate movement direction upon tactile contact (e.g., away from the contacted obstacle), the Material Classification unit provided information about the contacted obstacle.

On the wheeled robot, the antenna was actuated with a pan-tilt system controlled by the Motor Layer that mediated the information from the Control Layer. The current speed of the robot could be used to modulate the cycle frequency of the antennae in order to reduce possible shadow areas, thus avoiding collisions with obstacles.

Within the spiking network within the Sensory Layer shown in Figure 3, each unit is an Izhikevich-type spiking neuron (Izhikevich, 2003). The neuron model is represented by the following differential equations:

$$\begin{array}{c} \dot{v}\;=k\text{​}\left(0.04x^{2}+5v+140-u\;+I\right)\\ \dot{u}\;=ak\text{​}\left(bv-u\right) \end{array}$$

with the spike-resetting

$$\text{if }v\geq 0.03,\text{ then }\{\begin{array}{c} v\leftarrow c\\ u\leftarrow u+d \end{array}$$

where *v* is the membrane potential of the neuron, *u* is a recovery variable, and *I* is the synaptic current. By choosing the parameters *a*, *b*, *c*, and *d*, different kinds of neural dynamics can be obtained. To show a resonate-and-fire behavior the neuron parameters were set to the following standard values: *a* = 0.1, *b* = 0.26, *c* = −60 and *d* = −1. The parameter *k* was used to select the resonate frequency in each neuron. All other neurons behaved like class I neurons, in which the output spike rate was proportional to the input current (the adopted parameters were *a* = 0.02, *b* = −0.1, *c* = −55 and *d* = 6).

## Results

### Tactile contact localization

When the antenna hits an object, the acceleration sensor recorded the impact in the form of an abrupt, steep signal followed by a damped harmonic oscillation. The latter can be explained by the vibration of the free end of the probe, i.e., the part between the contact site and the tip. Accordingly, the fundamental frequency of the oscillation increased with increasingly distal contact events, i.e., with decreasing length of the vibrating free end of the probe (Figures 2B and C). The FFT amplitude spectra of the recorded oscillations always showed a salient low-frequency peak, followed by one or more high-frequency modes. In order to obtain good understanding of the main signal parameters and their relevance for the sensing process, we first limited the analysis to the two largest modes only, using their peak frequency, amplitude and decay time constant as parameters. Peak frequencies, *F*, of both modes increased exponentially with contact distance, *d* (Figure 2C, low frequency: log *F* = 0.0268 · *d* + 0.69; high frequency: log *F* = 0.0305 · *d* + 1.52), and linear regression models of the log-transformed frequencies explained more than 98% of the total variance of the data (low frequency: *r*^2^ = 0.995; high frequency: *r*^2^ = 0.987). Peak frequencies of the low-frequency peaks had approximately three times lower standard deviation than those of high-frequency peaks, indicating that the low-frequency peaks were more reliable for estimation of contact distance. Judged from the standard deviation of the low frequency peaks, the linear regression model allowed an average precision of 6.2 mm for estimates based on a single contact event.

**Figure 4:**
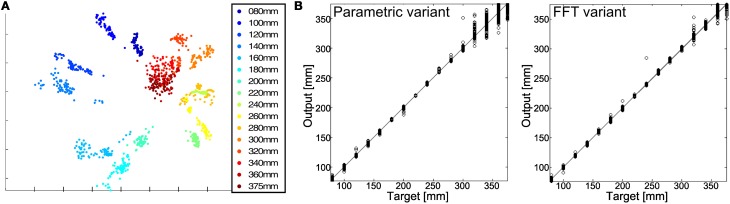
**(A)** Multi-dimensional scaling and tactile localization. The plot illustrates the separability of different distances. The Fourier spectra of eight materials with a contact distance from 80 mm to 375 mm were processed using multi-dimensional scaling. This led to a two-dimensional embedding with the pair-wise distances being conserved. Contact locations closer to the base can be distinguished easily (blue clusters do not overlap), while contact locations above about 320 mm are challenging to tell apart (red clusters overlap). Note that the scales on the axes are meaningless, as this visualization method uses a non-linear transformation with the sole purpose of conserving distances in a planar projection. **(B)** Corresponding confusion plots, showing the distance output of the artificial neural network compared to the true distance of the object. The results of parametric variant (left) are compared with those of the FFT-variant (right). The latter shows better results, especially for distant contact sites.

A major problem of this parametric variant of sensory processing was that the frequency of the first harmonics became increasingly unreliable for distal contact sites due to an decrease of the signal-to-noise-ratio. Nevertheless, we tested the performance of tactile localization with an ANN receiving two frequency peaks of the FFT spectrum as input, and producing a distance estimate at its output. For comparison, we used the entire amplitude spectrum of the FFT, thus increasing the input dimension from 4 to 509. The results are shown in Figure 4. The fact that vibration-based distance estimation of contact events generally worked well is reflected by the disjunct clusters of input vectors after multi-dimensional scaling (Figure 4A, note that multi-dimensional scaling is used for visualization purposes only, not for quantitative analysis). As yet, this graph reveals that clusters corresponding to increasingly distal contact locations overlapped more and more, suggesting that it should become increasingly hard for an ANN to separate the corresponding input vectors. In other words, since the network had to find a mapping from frequency values to distance, performance was expected to deteriorate for distal contact locations. The regression plots in Figure 4B confirm this. Compared to the precision estimate based on the linear regression models mentioned above, performance increased for both ANN variants. For the parametric peak search variant, the root mean squared error (rmse) of 2.93 mm, equivalent to 0.7% of the antenna length. For the FFT variant, performance improved to an rmse of 1.71 mm, equivalent to 0.4% of the antenna length. This improvement was not due to altered network size (owing to more input dimensions), because using the lower frequency band only (halving the input) yielded similar results as those of the parametric variant, whereas ten-fold sub-sampling the input spectrum (tenth input size) produced classification results that did not differ from those obtained from the entire spectrum. In summary, this shows that (i) the upper frequency band is important for good performance in vibration-based tactile localization, and (ii) that extraction of a single high-frequency mode is either not sufficient or not reliable enough for achieving the best performance in tactile localization.

### Tactile material classification

**Figure 5:**
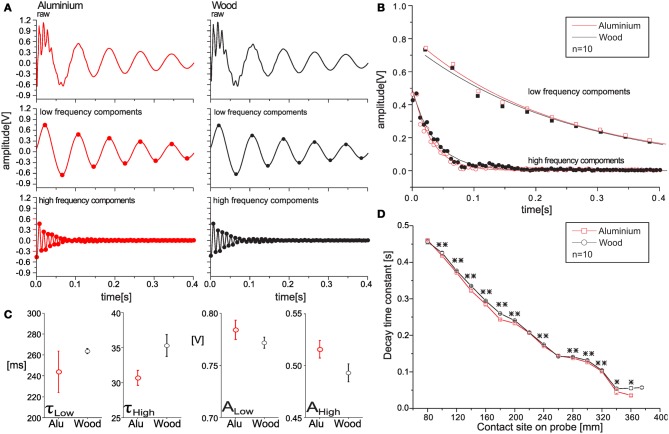
**The principle of tactile material classification.** Extracting parameters of damping. **(A)** When touching different objects or materials, like an aluminium rod (red) and a wooden rod (black), sensor readings hardly differ in frequency content (top), and even after decomposition, both low (mid panels) and high frequency components (bottom) look very much alike for both materials. **(B)** Extraction and rectification of local extremata of sensor reading reveals different time courses of the decay. **(C)** Fitting first order exponential decay functions to single trial data results in significantly different time constants, τ, and amplitudes, A, for both frequency components (*n* = 10, means ± s.d.). Hence, analysis of decay time constants allows material classification. Note that data in **(C)** correspond to a single contact distance. **(D)** Dependence of decay time constants on contact site. Two materials were compared, a soft wood rod (circles) and an aluminium rod (squares). Time constants were calculated from slopes of linear fits to log-transformed peaks of low-frequency components. Error bars depict the SD, asterisks label statistically significant differences (*n* = 10; ^**^p < 0.001; ^*^p < 0.05).

Apart from tactile localization of objects, we were interested in exploiting information arising through physical interaction of the contacting materials. For example we were hoping to distinguish different material-specific properties through differences in energy dissipation. In a first approach, we used the parametric variant of signal analysis and tested its applicability for tactile material classification. Figure 5 summarizes the proof of principle: the materials wood and aluminium could be distinguished reliably by comparing the decay time constants of the damped harmonic oscillations. This suggested that an ANN could be used for tactile classification of several materials, even if the signals recorded had identical peak frequencies (because of identical contact location on the probe), and very subtle differences in signal time course only (e.g., see time courses in Figure 5A). In our proof of principle experiment, extraction and rectification of the local extreme points revealed robust differences in decay time constants that could be measured reliably by fitting a first-order exponential decay function (Figure 5B). Amplitudes and time constants of the fit functions differed in a statistically significant manner for both the low- and high-frequency signal component (*t*-test; τ_low_: *t* = 3.140, *p* = 0.0057; τ_high_: *t* = 7.736, *p* < 0.001; *A*_low_: *t* = −3.683, *p* = 0.0017; *A*_high_: *t* = −5.934, *p* < 0.001; Figure 5C). A remarkable feature of the algorithm was the low variability of the results. For example, the coefficient of variation of the decay time constant of the low-frequency component, τ_low_, was less than 1%. Owing to the small variability, measurements from different materials could be distinguished with great reliability.

Potential problems of classification based on decay parameters could occur as decay time constants changed with frequency and, thus, contact distance. To test whether it was possible to distinguish time constants at any contact location, we compared signals recorded for contacts on wood and aluminium, varying contact location from 80 to 375 mm. For both materials, decay time constants decreased with contact distance. The dependence was almost linear (Figure 5D). Time constants of the two materials were statistically different at all but four contact sites. On the background of having shown that tactile distance estimation by an ANN could be very reliable, it was clear that the decrease of time constant as a function of contact location was possible too. This suggested that material classification independent of contact site should be possible for an ANN, using either the parametric or the FFT variant.

**Figure 6:**
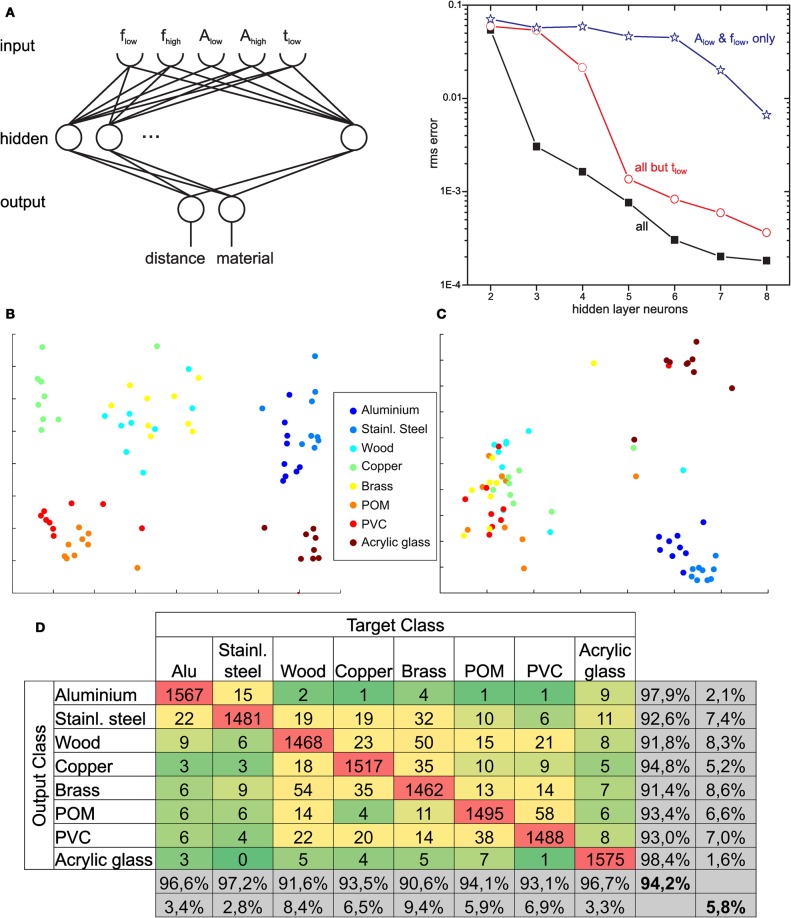
**ANNs for tactile material classification. (A)** In the parametric variant, a two-layered artificial neural network (ANN) was tested with different sets of input parameters for the high- and low-frequency components: extracted peak frequencies *f*_low_ and *f*_high_, amplitudes *A*_low_ and *A*_high_, and decay time constant of the low-frequency component, τ_low_. The diagram plots performance error over the number of hidden neurons for three combinations of input parameters (test error only, i.e., for signals not used for training). Lowest errors with smallest network size were obtained if τ_low_ was included as input. **(B)** and **(C)** illustrate the separability of different materials. The Fourier spectra of eight materials with a contact distance of 80 mm **(B)** and 320 mm **(C)** were graphed using multi-dimensional scaling, resulting in a two-dimensional embedding with pair-wise distances being conserved. **(D)** Confusion matrix, showing the output of the ANN compared to the true material class. As expected from multi-dimensional scaling, some materials could be distinguished easily, e.g., copper and aluminium, whereas others were more challenging, e.g., brass and wood.

In a first step of ANN-based material classification, we tested whether a two-layered perceptron could be trained to predict both the contact distance and the correct one of two materials (wood and aluminium). Figure 6A shows the network and the dependence of its performance on the number of hidden layer neurons. For identifying the most relevant input parameter for material classification, the ANN was trained with different sets of input parameters. If only the amplitude and frequency of the low-frequency component were used as input parameters, overall performance was bad (*blue line* in Figure 6A), mainly because errors in material classification were many. Performance improved if amplitude and frequency of the high-frequency component were included as well, but best performance (particularly for very small ANNs with few hidden neurons) was obtained if the decay time constant of the low-frequency component was included (*red* and *black lines* in Figure 6A, respectively). We did not include the decay time constant of the high frequency component because this was too unreliable to determine for distant contact locations where oscillation frequencies were very high (despite the fact that τ_high_ proved to be a reliable at proximal contact sites, as seen in Figure 5C). For the best ANN with five input, eight hidden and two output neurons (equivalent to 56 synaptic weights, only), the precision in distance estimation was ± 4 mm, with 87% correct material assignments for single contact events.

For testing the performance of material classification with several materials, we used eight cylindrical test rods made of different materials, including four metals and three plastics. Thus, the selection of materials included samples that were expected to be discriminated easily, e.g., aluminium and PVC, as well as samples that were expected to be much harder to distinguish, e.g., the two kinds of plastic. The experiments were carried out such that antennal contact occurred at 16 positions along the probe, ranging from 80 to 360 mm in steps of 20 mm, and at 375 mm. For each pair of material and contact location, contact events were recorded 100 times, yielding a total of 1600 sample measurements per material and 80 per contact distance. Multidimensional scaling of the input vectors for the FFT variant revealed that data points related to the same material clustered well, but with varying degree of overlap for selected material pairs (e.g., see brass and wood in Figure 6B). Moreover, overlap of clusters depended on contact location, as revealed by the different graphs for 80 mm and 320 mm in Figures 6B and C). When using the entire amplitude spectrum of the FFT as input, overall performance in material classification was very good, amounting to 94.2% of correct assignments for single contact events. As yet, performance was not equally good for all materials, as reflected by the confusion matrix in Figure 6D, where correct assignments are shown in red, low error numbers are shown in dark green, and increasingly larger error numbers are shown in light green and yellow. For example, wood and aluminium were confused only rarely, while brass and wood appeared to be more difficult to discern (brass was confused with another material in 9.4% of cases, wood was confused in 8.4% of cases; both of these materials were most likely confused with each other).

### Reducing network dimensionality while maintaining performance

**Figure 7:**
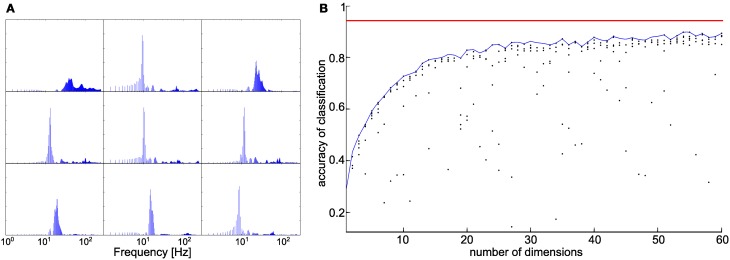
**NMF-based dimensionality reduction. (A)** Application of non-negative matrix factorization to the FFT spectra of contact signals lead to a set of basis vectors that could be interpreted as different band-pass filters. The nine frequency spectra correspond to the set of optimal filter characteristics when setting the dimension of the NMF-derived vector basis to nine. **(B)** Accuracy of material classification as a function of the number of dimensions of the NMF vector basis. The blue line delimits the maximum accuracy achieved for a given dimension. The red line indicates the accuracy achieved when using the original FFT spectrum as the input.

As the results above showed that the FFT variant was clearly superior to the parametric variant, the question remained which parts of the FFT spectrum were needed and which ones were not. As the FFT variant used 509 input dimensions, the resulting ANN was large, requiring considerably more computational resources than the parametric variant. Thus, there was a clear resource-performance trade-off, and we were interested to assess how performance decreased with systematic reduction of input dimension. As preliminary results had already suggested that ten-fold sub-sampling of the FFT spectrum had little effect on distance estimation (see above), we sought the most efficient combination in inputs in a data-driven manner. For this, we applied non-negative matrix factorization, NMF, with varying numbers of output dimensions. A representative result of NMF with nine dimensions is shown in Figure 7A. The number of nine dimensions was chosen for a better visualization, only. However, with a larger number of dimensions the shown band-pass filter like characteristics stays the same. The nine basis vectors are drawn as frequency spectra, emphasizing the analogy to a set of band-pass filters. The corresponding nine-dimensional input to the ANN was then computed by the dot products of the 509-dimensional FFT spectrum of the measured signal with each one of the basis vectors shown. Thus, the number of basis vectors is equal to the number of input dimensions of the ANN. Figure 7B shows how material classification improves with number of input dimensions, where several training sessions were done for each number of input dimensions, in order to avoid random effects. The performance when using the un-filtered, entire FFT spectrum was used as reference. The results for reduced input dimension asymptotically approached the results of the entire spectrum. For more than 30 input dimensions, performance in material classification was at least 86% correct assignments. From 30 onwards, the improvement of performance became smaller. Compared to the reference performance of 94.2% correct assignments with 509 inputs, the performance with 30 inputs was reduced by 8% only, while the amount of data was reduced by 95%. Taken together, robust classification of eight materials can be achieved by appropriately filtering the measured tactile contact signal by 30 non-linear band-pass filters obtained through NMF.

### Sensory data processing in spiking neural network

**Figure 8:**
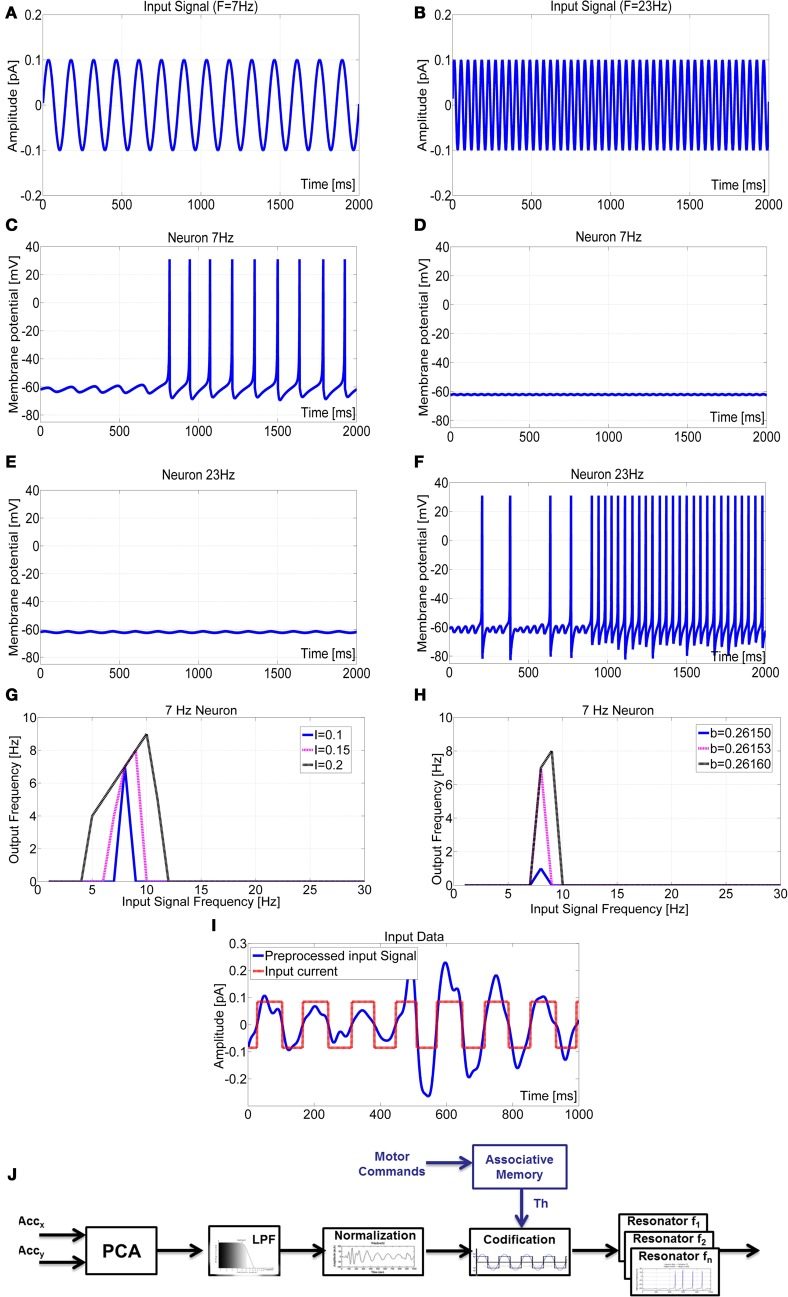
**Behavior of two neurons tuned to stimulus frequencies of 7 Hz (A) and 23 Hz (B).** Membrane potential of the 7 Hz neuron showed excitation in response to the sinusoidal stimulus with frequency 7 Hz **(C)** but no response to frequency 23 Hz **(D)**. The opposite was true for the 23 Hz neuron **(E)**,**(F)**. Tuning of frequency selectivity depended on parameters k and b, but also on the input amplitude I. This is illustrated for the 7 Hz neuron. Parameter *k* was used to select the frequency of interest (for 7 Hz: *k* = 0.3425). The input amplitude I modified the window in which the neuron was active **(G)**, and parameter *b* determined the spike rate **(H)**. **(I)** The pre-processed input signal was codified with a square-wave signal with an amplitude range [−0.085 to 0.085]. This improved the detection performance of the resonate neuron. **(J)** Block scheme of the signal elaboration from the sensory data acquisition to the neuron input current generation. An associative memory was considered to modulate the signal pre-processing depending on the robot motor command.

Until this point, the sensor data was always pre-processed by FFT. Although the sensory encoding of stimuli in the frequency domain is common in various sensory systems, including mechanoreceptive and visual systems, FFT is not a biologically plausible algorithm, so we strived to replace FFT by a bionic method of information processing. For this we used a frequency-encoding sensory array based on resonate-and-fire neurons. By appropriate parameter adjustment of a single resonate-and-fire neuron, it is possible to tune selective band-pass filters (Izhikevich, 2003). Here, we exploited this property to determine the specific frequency selectivity for each neuron in a linear sensory array, such that each neuron was responsive to a narrow band of frequencies only. The frequency tuning of the neurons is shown in Figure 8 where the responses of neurons with preferred frequencies at 7 Hz and 23 Hz are shown for two different sinusoidal input signals. Each neuron was responsive to the corresponding frequency and was silent to other inputs. The frequency selectivity is shown in Figures 8 G,H where the role of the parameters *I* and *b* was analysed for a neuron tuned to resonate at 7 Hz. By changing the amplitude of the input current, I, the neuron could either resonate in a wide range of frequencies or be exited in a narrow band around 7 Hz. Parameter b was responsible for the spike rate of the neuron.

After pre-processing with PCA, the signal was low-pass filtered to remove high-frequency disturbances (above 30 Hz). The data was then centred on zero by subtracting the mean value. This was done off-line but can be performed on-line too, e.g., by use of sliding window operations. Finally, the signal was transformed into a train of pulses of unit amplitude (Figure 8I), thus removing amplitude differences in the input to the resonate neurons that could affect frequency selectivity (which is a function of *I*, see Figure 8G). Among the different methods tested, this square-wave transformation proved most efficient, as the zero-crossings became triggering events for spikes. The result was a codification of input frequency only, supporting narrow-band frequency selectivity in each neuron within the sensory array.

### Spiking-based processing and forward model by sensory gating

Experiments carried out with the bionic antenna being mounted to the robotic platform generated a wide variety of sensor data that was used to test state-dependent modulation of sensory processing. During motion, frequency analysis of the pre-processed sensor readings revealed an evident peak around 7.8 Hz (note that high frequency signals with *f* > 30 Hz were not considered in this analysis). The presence of frequency components in the band 7–8 Hz is evident even in absence of tactile contacts. This activity represents the natural oscillation frequency of the sensory probe due to the robot/antenna motion. Still, antennal contact with an external object could be detected by a marked, impulsive response in the sensor reading. Upon registering a contact event, the antenna was maintained in contact with the obstacle for one second and a frequency analysis was performed. For this, the FFT-based analysis used so far was replaced by the spiking neural network architecture described in Figure 3, with appropriate parameter tuning as explained in Figure 8.

Analyzing the first results obtained using this pre-processing strategy, it was evident that, in some cases, low-amplitude fluctuations could create artifacts that erroneously caused a resonate neuron to fire a spike. To avoid this problem, a threshold for the signal amplitude was added, thus discarding sub-threshold fluctuations. The value of the threshold was critical because an inaccurate choice could lead to the presence of multiple false positives, or, on the contrary, to missing of all contact events (false negatives). Since most of the noise that made the threshold necessary was introduced by self-motion of the robot, this could be predicted from the motor commands assigned to the robot drives. In a first attempt to adaptively select an appropriate threshold value, we determined its dependence on robot speed. Indeed, if the robot drove on a plane surface, a simple speed-dependent threshold improved contact detection performance. Context-dependent setting of this threshold value allowed for state-dependent modulation of the sensitivity to contact events.

**Figure 9:**
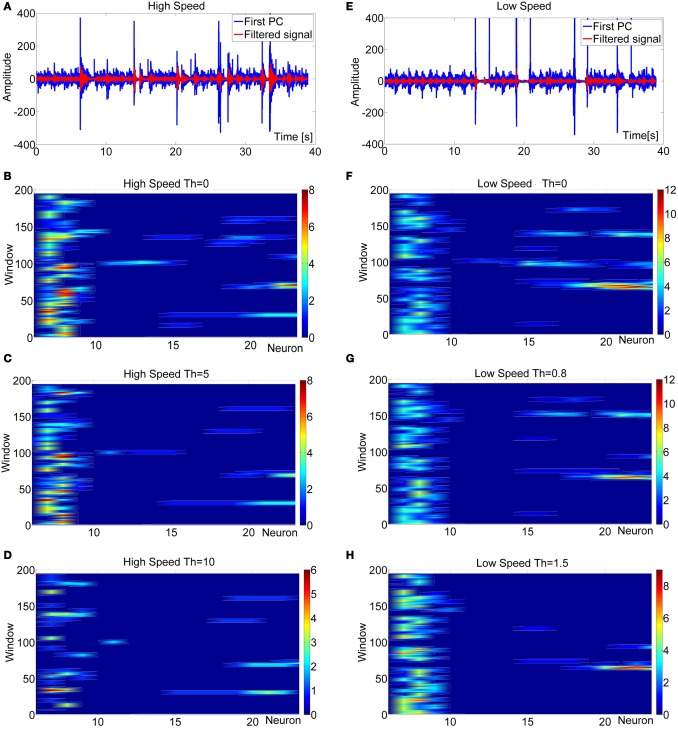
**Frequency maps obtained from data acquired during two experiments where the robot touched an obstacle several times and at different speeds: *v*_high_ = 27 cm/s, *v*_low_ = 12 cm/s.** In the principal component (PC) of the acceleration data, the contact events could be identified as high-amplitude impulsive responses. For the high-speed experiment **(A)** the contact events occurred at *t* = 6 s (window 30), *t* = 14 s (window 70), *t* = 21 s (window 105), *t* = 26s (window 130), *t* = 33 s (window 165). For the low-speed experiment the contact events occurred at *t* = 13 s (window 65), *t* = 19 s (window 95), *t* = 27 s (window 135), and *t* = 34 s (window 170). The maps show the responses of the sensory array of resonate neurons (columns of the maps), by color-coding their spike rate, in time. Rows correspond to time windows of 1 s duration, with a sliding time of 200 ms used between two consecutive windows. With a threshold *Th* = 0, some artifacts appeared as in **(B)** and **(F)** that do not correspond to real contact events. For high values of *Th*, real contact events could disappear from the map **(D)**–**(H)**. Finally, for optimal values of *Th*, contact events were detected correctly **(C)**–**(G)**. Note that the optimal value of *Th* depended on the speed of the robot.

The effect of threshold modulation on the sensory array of resonate neurons is shown in Figure 9. It summarizes two experiments in which the robot antenna touched obstacles several times. The rows in Figure 9 correspond to two different driving speeds (Low speed *v* = 12 cm/s; High speed *v* = 27 cm/s). When the signal codification through the square-wave was performed without a threshold modulation (*Th* = 0), contact events were detected erroneously, owing to the noise introduced by robot motion. Increasing the threshold can filter out disturbances, but too high thresholds can filter out contact signals, too. In the example shown, the optimal value of *Th* was strongly dependent on the speed of the robot, being more than five-fold as high for the fast speed than for the low speed. As yet, doubling *Th* led to a loss of true positives, irrespective of driving speed.

**Figure 10:**
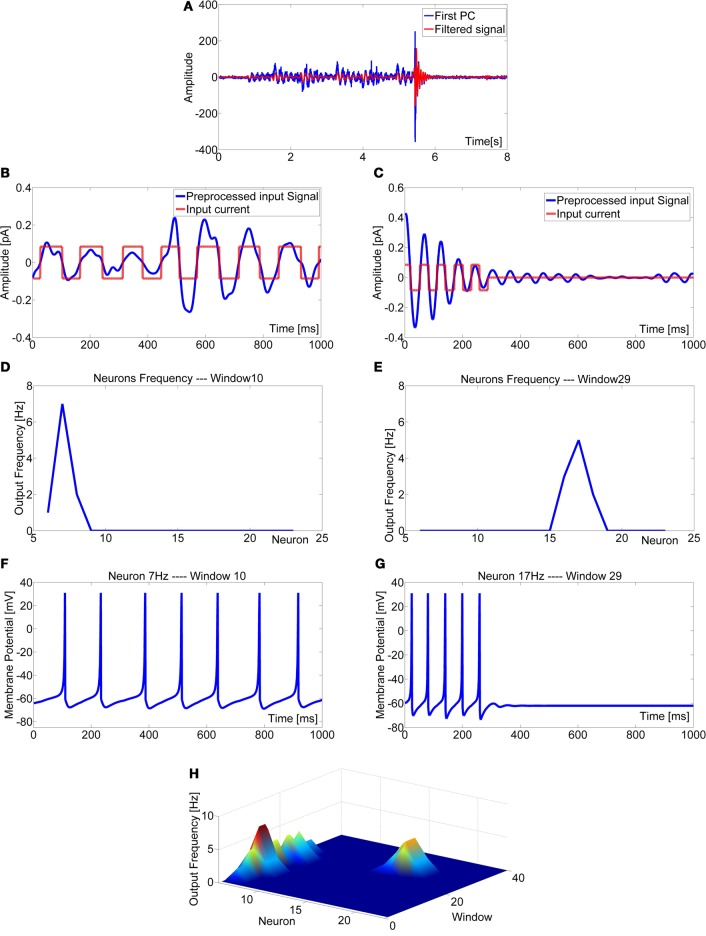
**Experiment performed with the robot exploring a flat terrain at low speed. (A)** Principal component extracted from the acceleration data and filtered signal (after low-pass-filtering with a cut-off frequency of 30 Hz). Behavior of the network in absence of contact events: **(B)** normalized signal in the interval [2, 3] s (window 10); **(D)** spike rate of the sensory array of resonate neurons: the 7 Hz neuron is active because the robot is in motion; **(F)** membrane potential of the 7 Hz neuron. Behavior of the network in presence of a contact event: **(C)** pre-processed input signal and corresponding input current in the interval [5.8, 6.8] s (window 29): a damped oscillation is recorded after a tactile contact; **(E)** spike rate of the sensory array of resonate neurons: the 17 Hz neuron it is active because the antenna touched an obstacle, the contact distance can be calculated using a non-linear mapping function (here *d* = 19 cm from the base of the antenna); **(G)** membrane potential of the 17 Hz neuron in window 29. **(H)** Frequency map obtained for the entire experiment.

A more detailed example of a robot experiment is shown in Figure 10, where the response properties of two resonate neurons in the sensory array during a non-contact episode (Figures 10 B,D,F) and a contact event (Figures 10 C,E,G) are juxtaposed. In this experiment, the robot was moving at low speed on flat terrain and a tactile contact occurred after 5.5 s. During self-motion without tactile contacts, the 7 Hz neuron fired spikes continuously, whereas neurons tuned to higher frequencies remained silent. Upon tactile contact with the obstacle, the pattern of activity within the sensory array shifted to higher frequencies, and the 17 Hz neuron was most active. The frequency map (Figure 10H) shows the time course of neural activity within the entire sensory array: the initial frequency band of 7–8 Hz indicates that the robot moved and, therefore, caused self-stimulation at the resonance frequency of the probe. The contact event was detected by an abrupt termination of the 7 Hz activity and simultaneous occurrence of a new peak at much higher frequencies. As soon as the robot was stopped in response to tactile contact, neural activity ceased in both high and low frequency neurons.

## Discussion

With this study, we present a bio-inspired tactile sensor for active tactile localization and material classification, suitable for application on a mobile robot platform. Two neural information processing modules were proposed for these purposes: a multi-layered perceptron for analysis of the frequency spectrum of the vibration signal recorded, and a spiking neural network that can provide the frequency spectrum and lends itself for state-dependent modulation of sensory processing during self-motion.

### Tactile localization and material classification

The measurement technique is simple, accurate, and robust. It is simple because the use of a single sensor per antenna, it is accurate because contact localization can achieve as little as 0.4% deviation from linearity over a measurement distance of some 40 cm, and it is robust because it allows for correct classification of eight materials in up to 94% of cases with a single contact event. Compared to earlier work on artificial active tactile sensors, our method differs with respect to the sensorization of the beam. For example Kaneko et al. (1998), Lewinger et al. (2005), Solomon and Hartmann (2006), and Pearson et al. (2011) used sensors at the base of the beam, whereas we use a sensor on the tip. Despite the difference in sensor placement, our method bears some similarities with that proposed by Ueno et al. (1998), who also inferred the contact location along the beam by analysing its resonant behavior, though with a basal sensor. Technically, a major difference between basal and distal sensor placement is the number of oscillation cycles that can be measured during a single contact event. In the approach by Ueno et al. (1998), the fundamental frequency during the contact period needs to be inferred from half a cycle period, whereas in our method, several cycle periods can be analysed. Analysis of signals containing several complete cycles of oscillation arguably allows for more reliable computation of the frequency composition. Another advantage of our method might be its applicability for material classification in addition to tactile localization. This was not tested by Ueno et al. (1998). On the other hand, approaches with basal bending- and/or torque-sensors have been shown to be very efficient in tactile shape reconstruction (e.g., Solomon and Hartmann, 2006). As our sensing method relies on the analysis of discrete events rather than of a continuous stream of sensor readings, it may be more appropriate for local, discontinuous analysis of object features rather than for complete and continuous mapping tasks.

The present system uses PCA-based pre-processing of the sensory input that reduces the number of input channels from two to one. This reduction is not necessary as the signals could be also processed separately, with the overhead of doubling the processing structure. An advantage of parallel processing of both channels might be that the direction of motion relative to the surface contacted could be determined. Future experiments will need to address this issue.

The major advantage of the feed-forward ANN module concerns the robustness of accurate localization in the face of noise, and the applicability of fast and simple learning rules for any particular tactile classification task of choice. Both of these aspects are of relevance to the engineering of autonomous active tactile sensing systems. Concerning the resource-performance trade-off, the reduction of input dimensionality used in this study was based on NMF, yielding a relatively small set of basis vectors for efficient description of the sensor signal. Owing to its non-negativity constraint, NMF results in a set of basis vectors that can be interpreted as non-linear band-pass filters. In principle, similar results might have been obtained by an even-spaced set of band-pass filters with equal frequency bandwidth. In fact, the analysis of distance estimation revealed that simple down-sampling of the input by using every 10th value, still produced satisfying results. Potential disadvantages of such “simpler” choices of sensory filters could arise due to arbitrary heuristics, e.g., when choosing the bandwidth of band-pass filters. In contrast, application of NMF ensured purely data-driven optimization of filter properties. The result suggests that some 30 band-pass filters provide sufficient information for reliable classification of the eight materials tested, independent of contact location. Many sensory systems are known to use a relatively small number of input channels with band-pass filter properties (e.g., see Olshausen and Field, 2004, on sparse coding in sensory systems). Thus, reducing the number of input dimensions by use of a set of band-pass filters is not only resource-efficient in technical terms, but also follows a common principle in natural sensory information processing.

Our active tactile sensor system is inspired by the stick insect antenna, albeit with strong simplification of the biomechanical features (Dirks and Dürr, 2011) and number of sensors (Monteforti et al., 2002). Stick insects of the species *Carausius morosus* continuously move their antennae during locomotion, thus actively exploring the ambient space (Dürr et al., 2001). They use mechanoreceptive information from their antennae for tactually mediated re-targeting of leg movements in a reach-to-grasp paradigm (Schütz and Dürr, 2011) though the source of this mechanoreceptive information is not known yet. At least two kinds of antennal mechanoreceptors could contribute to the encoding of fast rhythmic deformation of the long and thin flagellum: (i) campaniform sensilla, i.e., bending-sensitive sensilla that are embedded within the cuticle of different parts of the antenna; (ii) Johnston's organ, a prominent chordotonal organ in the second segment of the antenna, near the base. Johnston's organ is present in all higher insects. It is a proprioceptor known to measure antennal vibration in many insects, including mosquitoes, flies, and honeybees (reviewed by Staudacher et al., 2005). In stick insects, descending neurons of the antennal mechanosensory system have been shown to be vibration-sensitive (Westmark and Dürr, 2009), and the sensory structures that supply this information must have been located on the proximal part of the antenna, although their identity remains obscure. In a chordotonal organ of the stick insect leg, the sense of vibration has been analysed to considerable detail (Stein and Sauer, 1999), showing that the sense of vibration is of importance in these animals. In summary, the choice of a single distal acceleration sensor on our bionic antenna does not model any particular antennal mechanoreceptor. Rather it abstracts the property of antennal vibration-sensitivity, highlighting both advantages and disadvantages of vibration-sensitivity on an active sensor, and furthering our understanding of what kind of behavioral tasks vibration-sensitive proprioceptors potentially could contribute to.

In addition to their rich sensory infrastructure, real insect antennae have very complex mechanical properties. In many species, the antenna is sufficiently stiff for maintaining its shape during self-motion, even in very long and thin structures (e.g., 100:1 length-to-diameter ratio in the stick insect *Carausius morosus*). At the same time, antennae are compliant and readily bend when in contact with obstacles. Moreover, damping appears to be functionally important, and is known to vary along the length of the antenna (Dirks and Dürr, 2011). Near the base of the stick insect antenna, damping is over-critical, preventing long-lasting oscillation of the structure, while supporting fast return to the natural shape after release of contact. Closer to the tip of the antenna, damping is weaker and oscillation of the flagellum has been shown (Dirks and Dürr, 2011). The bionic tactile sensor used in this study does not model a proximal-to-distal gradient of damping properties. Taken together, the sensing principle based on a distal vibration sensor monitoring damped harmonic oscillations is a simplification based on the observation that antennal contacts cause deflections of the flagellum which lead to fast return movements inducing oscillation of the tip.

### State-dependent sensory gating by means of a forward model

Mechanoreceptors are particularly susceptible to self-stimulation during motion, mainly because movement may directly interfere with their adequate stimuli, e.g., the deflection of a hair or passive movement of a joint. As a consequence, active sensing, i.e., involving self-motion into the sensing process, is likely to generate self-stimulation that potentially could confound sensory readout. On the other hand, active mechanoreception may also have advantages in fidelity over passive mechanoreception, as shown by Kim and Möller (2006). Here we use a bio-inspired neural network approach for state-dependent modulation of sensory input. It is inspired by central modulation of sensory processing in insects (e.g., Poulet and Hedwig, 2007), and bears many parallels to gating properties described in the central nervous system of walking crickets (Staudacher and Schildberger, 1998).

Bio-inspired solutions can be applied in robots for finding alternative ways to deal with classical problems of obstacle detection in roving platforms. With the proposed spiking-network architecture we provide an alternative method for the frequency analysis of the input data originally performed through the FFT. The spiking-network is based on resonate-and-fire neurons for processing sensory information coming from mechanoreceptors on the antenna. Apart from resonance-based frequency decomposition of the input signal, we modulate a key parameter of the resonate-and-fire neurons for state-dependent modulation of their sensitivity. This allows for state-dependent cancellation of self-induced sensor readings.

Resonance in neural circuits is considered an essential ingredient for giving rise to self-sustained activity (i.e., in the absence of external stimuli), suggesting a primary role in higher cognitive processes such as working memory, decision-making, and goal directed behavior (Wang, 2003). On the other hand, dynamical system theory traditionally exploits resonance for detecting the essential dynamical characteristics of the system under consideration, emphasizing the role of enhancing specific input patterns locked around the system's resonance frequency. Joined together, these two concepts led to the design of neural models which were both able to exhibit self-sustained oscillations (Muresan and Savin, 2007), and to show the emergence of oscillations only if stimulated by input signals possessing specific frequency contents (Izhikevich, 2001). Complex strategies of selective communications were hypothesized using networks endowed with such models (Izhikevich et al., 2003). One of the first applications of resonators was in the field of sound detection, localization and clustering from sensory data (Arena et al., 2005). A similar principle has been applied by Webb et al. (2007), investigating the ability of bushcrickets to respond to different song patterns. The implementation through spiking networks, as proposed here, can be a first step toward a bio-inspired formalization of the structure, allowing its transfer and application to modeling higher brain functions of insects as well, for example functions that involve the mushroom bodies or the central complex (Arena et al., 2010). Future work will consider the introduction of local excitatory and global inhibitory connections among the resonate neurons to create a winner-takes-all topology to improve the filtering capabilities and the detection performance. Moreover the proposed architecture allows embedded solutions by using either networks of microcontrollers or FPGA-based boards (Arena et al., 2007, 2008).

In nature, mechanisms for self-motion detection are frequently met. For example, flies readily estimate their self-motion from the acquired optic flow field (Krapp, 2009). Forward models are used for predicting and compensating specific effects of own body motions, recorded from exteroceptive sensors: there is a large body of literature suggesting that biological systems use efference copy and internal models to filter out disturbances in a fast, robust, and adaptive way (Franz et al., 2004; Webb, 2004). Applications in the area of biorobotics include prediction and compensation of self-induced disturbances on sensor readings in a biped robot (Manoonpong and Woergoetter, 2009), using a recurrent neural network. The particular implementation of forward models through spiking neural networks is an interesting topic as discussed by Russo et al. (2005). Their modeling work on multimodal integration in crickets allowed them to deal with conflicting effects of auditory and visual orientation reflexes (phonotaxis and optomotor turning response). For this, they suppressed self-induced visual input activity during voluntary turning movements and phonotaxis-induced turning. In this case, a simple non-linear feed-forward compensator was designed as part of a bio-inspired spiking neural network to model sensorimotor integration and control on a roving robot. The proposed architecture included a forward model for ego-motion compensation that was based on a sensory gating strategy for an efficient and robust solution, requiring neither time prediction nor compensation mechanisms. The present model could be extended to include a reward-based learning method, allowing the robot to learn the appropriate threshold values. A potential candidate structure for implementing such a learning method is a Motor Map (Ritter et al., 1992). In a Motor Map, a lattice of neurons can be specialized to find the best threshold value for different robot speed. Moreover, it could be extended easily to include further inputs, including inputs that classify different types of environments. The reward signal could be generated by a teacher that indicated to the robot whether a detected contact event was a true or false positive. Instead of a teacher, other sensors, such as sonar or infrared distance sensors, could provide the signals necessary validation of true positives.

The control architecture used in the present robot system can be improved further by introducing a behaviour association network as proposed in Arena et al. (2009). The robot could then try different basic behaviors on the detected objects, for instance avoidance, climbing, or pushing, while monitoring the consequences of its own actions. By using a simple associative learning method, the robot could choose the most suitable action for each object, depending on information about dimension and type of material that can be acquired from the bionic antennae. It is important to underline that the spiking-network structure can be considered as a functional module that can be integrated with other modules for a more detailed control strategy.

In summary, out active tactile sensing system successfully applies a simple feed-forward ANN module for robust and reliable tactile localization and material classification. Moreover, we exploit the resonant behavior of a spiking-network for implementing a mechanism of state-dependent modulation or gating. This allows suppression of self-induced mechanorecptive inputs from the antenna as it occurs during self-motion. Thus, it is applicable to active tactile sensors mounted to mobile platforms.

### Conflict of interest statement

The authors declare that the research was conducted in the absence of any commercial or financial relationships that could be construed as a potential conflict of interest.
